# Decoupling Action Potential Bias from Cortical Local Field Potentials

**DOI:** 10.1155/2010/393019

**Published:** 2010-02-03

**Authors:** Stephen V. David, Nicolas Malaval, Shihab A. Shamma

**Affiliations:** ^1^Institute for Systems Research, University of Maryland, College Park, MD 20742, USA; ^2^National Institute for Applied Sciences, 69100 Villeurbanne, France

## Abstract

Neurophysiologists have recently become interested in studying neuronal population activity through local field potential (LFP) recordings during experiments that also record the activity of single neurons. This experimental approach differs from early LFP studies because it uses high impendence electrodes that can also isolate single neuron activity. A possible complication for such studies is that the synaptic potentials and action potentials of the small subset of isolated neurons may contribute disproportionately to the LFP signal, biasing activity in the larger nearby neuronal population to appear synchronous and cotuned with these neurons. To address this problem, we used linear filtering techniques to remove features correlated with spike events from LFP recordings. This filtering procedure can be applied for well-isolated single units or multiunit activity. We illustrate the effects of this correction in simulation and on spike data recorded from primary auditory cortex. We find that local spiking activity can explain a significant portion of LFP power at most recording sites and demonstrate that removing the spike-correlated component can affect measurements of auditory tuning of the LFP.

## 1. Introduction

The local field potential (LFP) is the integrated electrical activity of a large number of anatomically neighboring neurons, reflecting a combination of synchronous synaptic potentials [1] and action potentials [2]. The LFP is the object of growing interest in the neuroscience community because it may provide a valuable link between single neuron recordings and larger-scale neurophysiological signals such as EEG [3], fMRI [2], and ECoG [4]. These latter signals also offer a means to measure synchronous neural activity both within a single brain area [5, 6] and between brain areas [7, 8].

Historically, methods for studying LFP were developed with low impedance electrodes that integrated electrical potentials over a large brain volume (<1 MΩ, [1]). During the recent resurgence of interest in LFP, most studies have focused on data acquired with high impedance electrodes (1–5 MΩ) that can isolate the activity of single neurons. Single- or multiunit activity is typically extracted from the voltage trace by high-pass filtering (>∼600 Hz, [6, 9]), and the LFP is extracted by low-pass filtering the same signal (<~300 Hz, [5, 6, 10]). Little is known about how the high impedance and specialized tip geometry of electrodes used for single unit recordings affect LFP signals in the lower frequency band. Thus, it is possible that the nature of the LFP signal from high impedance electrodes differs from classical low impedance recordings.

Several recent studies have compared sensory tuning of spiking activity with different bands of the LFP signal in auditory [6, 11] and visual cortex [10]. These studies have suggested that the tuning of high frequency LFP signals is correlated with the tuning of single units recorded at the same site. However, little attention has been paid to the possibility that activity from spiking events might overlap with the LFP signal, especially at the upper end of the LFP band. High frequency (spikes) and low frequency (LFP) bands are technically orthogonal. However, spike events and the excitatory postsynaptic potentials (EPSPs) that immediately precede them can contribute power to both bands. If the spiking/EPSP events of nearby neurons do contribute significantly to the LFP signal, then what appears to be a strong correlation between nearby single unit activity and broader population activity in the LFP may instead be an artifact of the single unit activity that survives low pass filtering.

To address this problem, we have developed a method for identifying components of the low frequency LFP band that can be predicted by spiking events. This procedure uses standard linear systems identification methods [12] to correlate activity between spiking events in the high frequency band with activity in the LFP band independent of any stimulus or behavior events. Components of the LFP that can be predicted directly from spikes are removed from the raw signal to produce a “clean” LFP. This simple procedure can be applied to any simultaneous spike and LFP recording.

In this study we first use simulated electrophysiological signals to evaluate the feasibility of the procedure for removing features correlated with spike activity from LFP signals. We then applied the procedure to recordings from primary auditory cortex (A1) of passively listening ferrets. We tested several different definitions of spike events in the electrophysiological recordings. For conservative (i.e., high threshold) definitions, the contribution to LFP power was relatively small but often significant. More liberal definitions of multiple single unit activity or multiunit activity explained a larger portion of the LFP signal. Even for the most conservative definition of spiking, however, we found that removing the spike-coupled component could change the sensory tuning of the LFP.

## 2. Methods

### 2.1. Experimental Procedure

Extracellular electrophysiological activity was recorded from primary auditory cortex (A1) of three awake, passively listening ferrets. All experimental procedures conformed to standards specified by the National Institutes of Health and the University of Maryland Animal Care and Use Committee. The basic experimental methods have been reported in detail previously [13].

#### 2.1.1. Surgical Preparation

Animals were implanted with a steel head post to allow for stable recording. While under anesthesia (ketamine and isoflurane), the skin and muscles on the top of the head were retracted from the central 4 cm diameter of skull. Several titanium set screws were attached to the skull, a custom metal post was glued on the midline, and the entire site was covered with bone cement. After surgery, the skin around the implant was allowed to heal. Analgesics and antibiotics were administered under veterinary supervision until recovery.

After recovery from surgery, a small craniotomy (1-2 mm diameter) was opened through the cement and skull over auditory cortex. The craniotomy site was cleaned daily to prevent infection. After recordings were completed in one hemisphere, the site was closed with a thin layer of bone cement, and the same procedure was repeated in the other hemisphere.

#### 2.1.2. Neurophysiology

Single unit activity was recorded using tungsten microelectrodes (1–5 MΩ, FHC, Bowdoin, ME) from head-fixed animals in a double-walled sound-attenuating chamber (Industrial Acoustics Company, Bronx, NY). During each recording session, one to four electrodes were positioned by independent microdrives, and activity was recorded using a commercial data acquisition system (Alpha-Omega, Alpharetta, GA). For most recordings, a 60 Hz notch filter was used to remove ambient noise. Because low frequency components of extracellular recordings tend to contain substantially more power than high frequency components, the analog signal was filtered into low (1–1000 Hz) and high frequency (300–6000 Hz) bands before digitization. The low frequency band was digitized with a 3125 Hz sampling rate, and the high frequency band was digitized with a 25000 Hz sampling rate. The separation of spikes and LFP frequency bands is a standard procedure used by commercial data acquisition systems and outside of experimental control. The analysis described below could also be performed on a single signal, appropriately band-pass filtered to extract spike and LFP bands. 

Upon identification of a recording site with single units, a sequence of random tones (100 ms duration, 500 ms separation) was used to measure latency, and spectral tuning. Neurons were verified as being in A1 according to by their tonotopic organization, latency and simple frequency tuning [14].

### 2.2. Stimuli

Stimuli were narrowband noise bursts. Center frequency was sampled logarithmically from 500 to 16000 Hz and bandwidth was scaled with center frequency so that each burst had a fixed width in octaves (0.125–0.25 oct, 20–40 noise bursts total). Each burst was generated by summing 20 pure tones at random phase, logarithmically spaced between the minimum and maximum frequency. Each noise burst was presented for 1.5 seconds with 0.8 second interstimulus intervals, and the entire set was presented for 10 repetitions. Thus the total length of data recorded from a given site was 460–920 seconds, depending on the number of distinct stimuli.

Stimuli were presented from digital recordings using custom software (Matlab, Natick, MA). The digital signals were transformed to analog (National Instruments, Austin, TX), equalized to achieve flat gain (Rane, Mukilteo, WA), amplified to a calibrated level (Rane, Mukilteo, WA) and attenuated (Hewlitt Packard, Palo Alto, CA) to the desired sound level. These signals were presented through an earphone (Etymotics, Elk Grove Village, IL) contralateral to the recording site. Before each experiment, the equalizer was calibrated according to the acoustical properties of the earphone insertion. All stimuli were presented at the same sound level during a single experiment (65–75 dB SPL, varying between recording sites).

### 2.3. Analysis

Spiking activity was extracted from the high frequency (300–6000 Hz) component of the recorded electrophysiological signal, *r*_*h*_(*t*), using two different methods. For the first, multiple single unit spiking activity (SUA) was identified by the time when the recorded potential underwent a rapid decrease during a single time step (1/25000 Hz  = 0.04 ms)

(1) $$s_{\text{SUA}n}\left(t\right)=\{\begin{array}{cc} 1, & r_{h}\left(t\right)-r_{h}\left(t-1\right)<-n\sigma ,\\ 0, & \text{otherwise}, \end{array}$$

so that nonzero values of *s*_SUA*n*_(*t*) indicated the likely occurrence of a spike at time *t* if the decrease from the previous time bin was greater than a threshold, *n* *σ*. The value of *σ* was the standard deviation of *r*_*h*_(*t*), and *n* was a scaling term that specified a sensitivity threshold. This thresholding procedure is a common first step in spike sorting algorithms [15]. After threshold spiking events were identified, the events were binned at 300 Hz, to match the sampling rate of the LFP signal (see below).

The specific choice of threshold (or even the definition of spike events) may vary across experiments, the logical choice being the threshold used for spike sorting (in previous studies using the same experimental procedures, *n* = 4 was used, [15]). Smaller values of *n* are more permissive and identify a larger number of spiking events while larger values are more conservative. We explored several possible values of *n*, but in this study data we report data for *n* = 3 or 4, which capture the essential variability resulting from different thresholds. In some brain regions, spike events might be identified by positive, rather than negative, changes in potential. In the experiments reported here, a negative potential change always detected spike events more reliably, but any alternative definition of spiking events can be substituted at this stage of the procedure without changing the subsequent steps.

The second method of measuring spiking activity used a common definition of multiunit activity (MUA). Rather than identifying single spike events, the activity of multiple neighboring neurons was approximated by squaring and low-pass filtering *r*_*h*_(*t*) [9]

(2) $$s_{\text{MUA}}\left(t\right)=\sqrt{{\text{LP}}_{150}*{\left({\text{LP}}_{3000}*r_{h}\left(t\right)\right)}^{2}}.$$

Here, *“*LP_*f*_ ∗” indicates convolving with a low-pass filter with cutoff frequency *f*, and *s*_MUA_(*t*) indicates the relative multiunit activity at time *t*. For this study, linear-phase finite impulse response (FIR) filters of order 500 (duration 500/25000  = 20 ms) were used. The final low-pass filtering at 150 Hz allowed downsampling the MUA signal to 300 Hz without aliasing artifacts. We compared this definition of MUA to another definition [6] and did not observed any qualitative differences other than a slight improvement in signal-to-noise in the spike-LFP filter for the definition in (2).

The raw local field potential (LFP), *L*_0_(*t*), was extracted from the electrophysiological recording by low-pass filtering (<150 Hz, linear-phase FIR, duration 100 ms) of the low frequency component of the recorded electrophysiological signal [6, 10, 11]. The signals *L*_0_(*t*) and *r*_*h*_(*t*) existed in entirely different frequency bands and thus were orthogonal (i.e., linearly uncorrelated). However, extracting single or multiunit activity from *r*_*h*_(*t*) involved nonlinear computations that could reintroduce linear correlation between them. This coupled component was identified by measuring their cross covariance

(3) $$c_{sl}\left(\tau \right)={\langle \left(L_{0}\left(t\right)-{\langle L_{0}\rangle }_{t}\right)\left(s\left(t-\tau \right)-{\langle s\rangle }_{t}\right)\rangle }_{t}.$$

The spike signal used here, *s*(*t*), could be any of the different spike signals defined above. In this study, *c*_*sl*_, *c*_*ss*_, and *h* (see below) were estimated for *τ* = −500,…, 500 ms. Larger values of *τ* had no effect on filter estimates (note width of nonzero filter range <200 ms in Figures 2 and 3).

In order to remove all spike-coupled features from the LFP signal, we generated a filter that made the best (i.e., minimum mean-squared error) prediction of the LFP from the spike signal. The filter was estimated by standard linear regression [12] and assumed that the spike-LFP relationship was stationary throughout the recorded data set. First, the autocovariance of the spike signal was measured

(4) $$c_{ss}\left(\tau \right)={\langle \left(s\left(t\right)-{\langle s\rangle }_{t}\right)\left(s\left(t-\tau \right)-{\langle s\rangle }_{t}\right)\rangle }_{t}.$$

The filter was then computed by division in the Fourier domain

(5) $$\hat{h}\left(\omega \right)=\frac{{\hat{c}}_{sl}\left(\omega \right)}{{\hat{c}}_{ss}\left(\omega \right)},$$

where *h*(*τ*) was the final filter and the hat symbol (e.g., $\hat{h}\left(\omega \right)$), indicates a Fourier transform of the corresponding time-domain function. To remove edge artifacts, a Hanning window of the same length as *h* was applied to *h*(*τ*), and the mean was subtracted to remove any DC bias introduced by the Hanning window. 

To remove the spike-coupled components from the LFP signal, the spike-LFP filter was convolved with the spike signal to predict the LFP

(6) $$L_{\text{pred}}\left(t\right)=\overset{}{\underset{\tau }{\sum }}s\left(t-\tau \right)h\left(\tau \right),$$

and the prediction was subtracted from the raw LFP signal to produce a signal with no correlation with local spiking activity,

(7) $$L\left(t\right)=L_{0}\left(t\right)-L_{\text{pred}}\left(t\right).$$

To distinguish the effects of the different spike identification algorithms, each cleaned LFP signal that was labeled with a subscript to identify the definition of the spike signal used for cleaning (e.g., *L*_SUA4_(*t*) was the LFP signal cleaned of correlations with *s*_SUA4_(t)). Note that this procedure models the LFP as a linear sum of large-scale and locally coupled activity, and any nonlinear interactions between them will persist in the cleaned LFP signal.

Matlab code demonstrating this procedure is available online (http://www.isr.umd.edu/Labs/NSL/Download/infer_spikes_LFP.m). Documentation is included in the code's comments.

### 2.4. Validation with Simulated Electrophysiological Recordings

**Figure 1:**
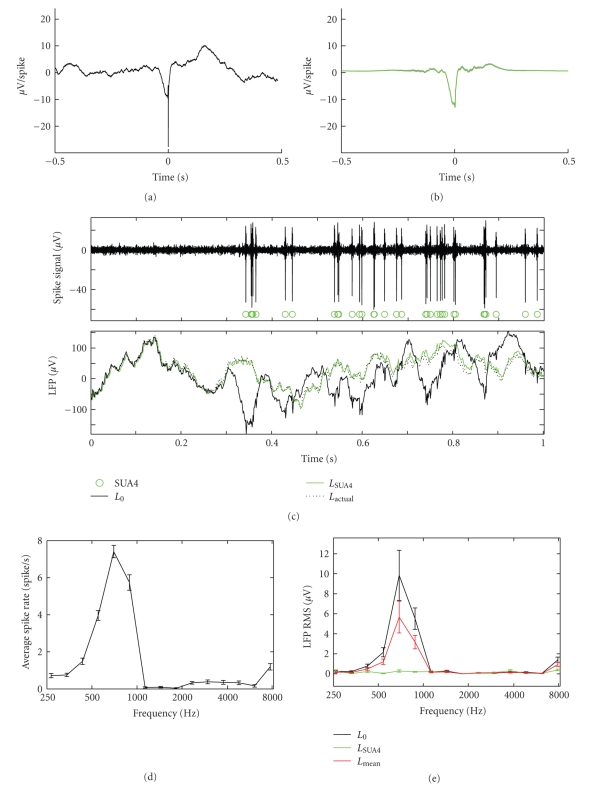
Removal of spike-coupled LFP signal in simulated data. (a) Average waveform of simulated frequency-tuned spiking activity introduced to a random (1/*f* noise) LFP signal. (b) Spike-LFP filter estimated by (5). (c) (Top panel) High-frequency spike signal, *r*_*h*_(*t*), with SUA4 events marked by green circles. (Bottom panel) Raw LFP signal (*L*_0_, black) and “clean” LFP signal with SUA4 events removed using filter in (b) (*L*_SUA4_, green). The clean LFP closely matches the underlying LFP signal (*L*_actual_, black dotted line) before spiking activity was added. (d) Frequency tuning of SUA4 activity. (e) Frequency tuning measured for raw LFP shows tuning similar to spiking activity (black). After SUA4 activity was removed, frequency tuning disappears (green). When only the mean spike-LFP correlation is subtracted for each spike event, the tuning is only partially removed from the LFP signal (red).

In order to test the algorithm for removal of spike-correlated activity from the LFP signal, we simulated the activity of a site in A1, using the same band-pass noise stimuli as the actual physiology experiments. Spiking activity was simulated by applying a linear spectrotemporal filter ([13], best frequency 600 Hz) to the stimulus spectrogram. Spike times were then generated by passing the rectified output of the linear filter through a Poisson spike generator for 10 repeated stimulus presentations. An average spike waveform (Figure 1(a)) was generated by averaging spike events in a broad-band (5–12000 Hz) physiological recording from A1. For each spike event, this waveform was added to a random LFP signal (1/*f* noise). Thus the “raw” LFP signal would appear to have tuning, due to the addition of the low frequency component accompanying each action potential, while the “clean” LFP signal should not.

The SUA4 analysis was applied to the simulated data to illustrate the effects of removing spike-correlated activity from the LFP. The estimated filter (Figure 1(b)) showed a strong resemblance to the spike waveform, except for the absence of the very rapid depolarization at time 0 that falls outside the frequency range of the LFP signal. The effects of removing spike-correlated activity are illustrated in Figure 1(c). As dictated by the simulation, the raw LFP (solid black line) deviated from the original (1/*f* noise) LFP (dashed black line) during periods of spiking activity. When the signal predicted by the spike events was removed (green line), the LFP closely matched the original. Concordantly, the raw LFP signal (Figure 1(e), black line) showed similar frequency tuning to that of the spiking events (Figure 1(d)). When the spike-correlated events were removed, the tuning disappeared (Figure 1(e), green line). Note also that the slight suppression in the spike tuning between 1000 and 2000 Hz did not appear even in the raw LFP tuning. The lack of suppressive tuning reflects the fact that the absence of spikes has no effect on the LFP signal in this model of spike-LFP interactions.

To compare the current method with a published method for removing action potential artifacts from the LFP, we applied an alternative artifact removal algorithm to the same simulated data [16]. This method computed the average LFP waveform associated with each spiking event (i.e., the cross correlation in (3)) and subtracted that average from the LFP at the time of each spike. Subtracting the mean waveform did not completely remove auditory tuning from the LFP signal (Figure 1(e), red line). This incomplete cleaning is likely due to the fact that the mean subtraction method does not account for the autocorrelation of the spiking activity (5), which is required to achieve a minimum mean-squared error estimate of the spike-LFP filter [12].

### 2.5. Significance Testing

A cross-validation procedure was used to avoid overfitting of spike-LFP filters. The data was divided into 20 segments of equal size. For each segment, the applied filter was estimated from the remaining 19 segments. Thus the data used to fit the filter (5) was independent of the data to which the filter was applied (6).

**Figure 2:**
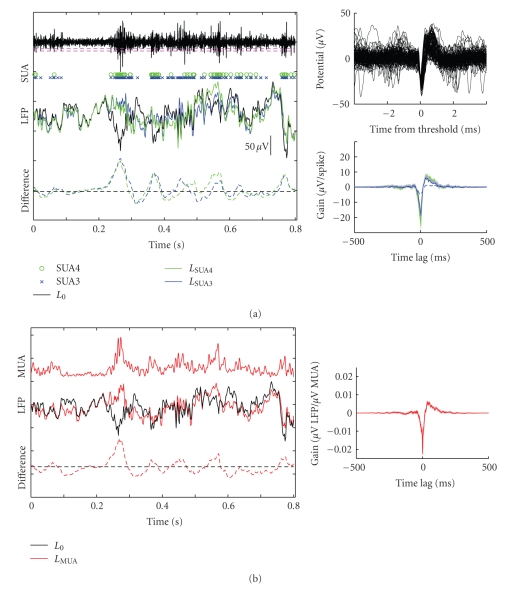
Example spike and LFP responses for an electrophysiological recording from primary auditory cortex (A1). (a) Brief segment of a raw high-pass filtered signal (black curve, top) and spike events identified by sudden changes in the signal (SUA*n*, circles, and “*x*”s). Green and blue dashed lines indicate, respectively, the thresholds for SUA4 and SUA3 events. Subpanels at right show 100 examples of SUA4 spikes events (a) and the impulse response function that best predicts the LFP (b) from SUA4 (green) or SUA3 events (blue), with standard error indicated by the shading. The dashed blue line shows the SUA3 filter estimate for spike events on a second electrode 0.4 mm from the LFP electrode. The simultaneously recorded raw LFP (black curve, middle) was substantially modified when the component predicted by SUA4 or SUA3 events was removed. The difference between the cleaned and raw LFP signals was the greatest during periods of elevated spiking activity (dashed curves, bottom). (b) The same procedure for removing coupled spike information from the LFP but using multiunit activity (MUA). The MUA signal for the same data segment as in *A*, defined by (2), captured the elevated firing at 0.3 second (red curve, top). The LFP signal with MUA removed (red curve, middle; difference in dashed line, bottom) roughly followed the same pattern as the LFP with SUA removed. The subpanel at right shows the impulse response that best predicted the LFP from the MUA.

**Figure 3:**
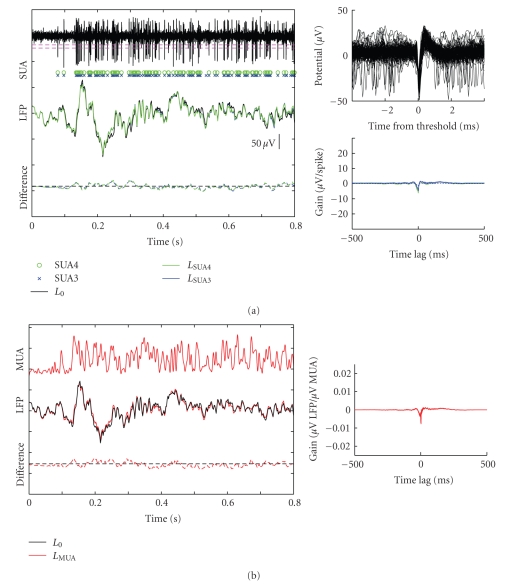
Example spike and LFP responses for a second recording site. Data are plotted as in Figure 2. (a) The impulse responses for SUA4 and SUA3 (subpanel at lower right) are smaller than the impulse responses in Figure 2, and using this function to remove LFP components that could be predicted by SUA events had very little effect. (b) Similarly, removing LFP components that could be predicted by MUA had very little effect on the LFP for this site.

**Figure 4:**
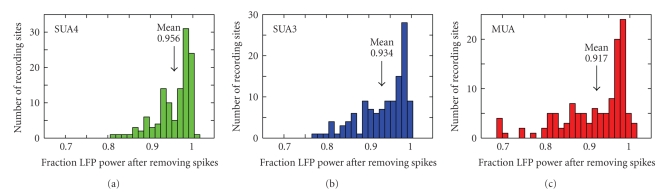
Effect of removing coupled spike activity on total LFP power. (a) Histogram of the ratio of power in the LFP after removing components explained by SUA4 and power in the raw LFP (*n* = 127 recording sites). For a small number of sites, LFP power increased slightly, reflecting the introduction of a small amount of noise by the cross-validation procedure used for filter estimation. (b) Histogram of change in power after removing the SUA3 component. The average power was significantly lower than for SUA4 (jackknifed *t*-test, *P* = .0008). (c) Histogram of change in power after removing the MUA component. The average power was significantly lower than for SUA3 (jackknifed *t*-test, *P* = .0007).

Standard errors on spike-LFP filters (Figures 2 and 3) were estimated by jackknifing [17]. This method allows unbiased significance tests for differences between random variables with non-Gaussian distributions, such as those often encountered in neural data. Significant changes in the LFP signal across the population of recording sites due to the removal of spike-correlated activity (Figure 4) were tested by a jackknifed *t*-test based on the same method of standard error estimation [17].

## 3. Results

Electrophysiological activity was recorded with high impedance tungsten electrodes (1–5 MΩ) from primary auditory cortex (A1) of awake ferrets. Neuronal action potentials and the local field potential (LFP) were measured from activity in high (300–6000 Hz) and low (1–300 Hz) frequency bands, respectively, of the raw electrophysiological trace (see Methods for details). Band-pass noise stimuli were presented during passive listening to measure auditory tuning properties of the single unit and LFP signals (460–920 seconds of data per site). 

Most studies use a standard definition for LFP [5, 6], but several different methods have been developed for measuring spiking activity. Some of these different methods are illustrated for a brief segment of raw data in Figure 2. The top of Figure 2(a) shows the high-pass filtered electrophysiological trace. In the first method, SUA4, spike events from single units near the electrode were defined as a sudden decrease in potential greater than four times the standard deviation of the high-pass signal (see (1), *n* = 4, green circles, Figure 2(a)). A less conservative method, SUA3, required a decrease of only three times the standard deviation, thus identifying a larger number of spike events, probably from a larger number of neurons (see (1), *n* = 3, blue “*x*”s). Finally, the least conservative method measured multiunit activity (MUA) by low-pass filtering the power in the electrophysiological trace (see (2), [9]). The MUA measurement produced a continuous signal that approximated the activity of a larger group of neurons near the electrode tip (red trace, Figure 2(b)).

In order to measure the component of the raw LFP signal that could be directly predicted by spiking events, we measured the impulse response between spike activity (defined using each of the spike models described above) and the LFP signal. The impulse response was measured as the cross covariance between spikes and LFP, normalized (i.e., deconvolved) by the autocorrelation of the spike activity (see (5), [12]). When convolved with the spike signal, the impulse response acted as a filter that produced the minimum mean-square error estimate of the LFP. The predicted response was subtracted from the raw LFP (7) to produce a “clean” version of the LFP with spike-coupled information removed.

Impulse response functions measured for the example recording site using the SUA4 and SUA3 signals appear in the bottom right panel in Figure 2(a), and the impulse response for MUA appears in the inset in Figure 2(b). If there were no correlation between spiking activity and the LFP, this function would be flat. Instead, in all three cases, the impulse response had a highly significant structure (note small error bars indicated by shading) (Figure 2(a), top). The significantly nonzero filter indicates that low frequency voltage changes (spike-related activity and EPSPs) were in fact correlated with action potentials and survived the low-pass filter into the LFP signal. These filters have shapes typical of filters measured across the entire set of recording sites studied, featuring a depolarization about 100 ms in duration around the time of the spike. The impulse response for the high threshold definition of single unit activity (SUA4) had slightly larger amplitude than the impulse response for lower threshold (SUA3) but its shape was nearly identical. A smaller magnitude filter was estimated when the SUA3 signal was measured simultaneously from an electrode 0.4 mm away (dashed blue line), indicating that local neuronal activity was responsible for the correlation between spike and LFP signals. The MUA impulse response also had a similar shape to the SUA impulse responses. Given the difference in units used for SUA and MUA, a direct comparison of filter magnitudes is difficult, although the magnitude of their effect on LFP signals can be compared (see below).

The middle of Figure 2(a) shows the result of subtracting the component of the LFP that could be predicted by SUA4 (green) or SUA3 (blue) from the raw signal (*L*_0_, black). The change between the raw and cleaned LFP was the greatest during periods of elevated spiking firing (e.g., at time 0.3 second). The difference between the raw and clean LFP is plotted in the dashed curves at the bottom of Figure 2(a). Overall, the more liberal definition of spike events had a greater effect on the LFP signal. The LFP cleaned by the SUA4 signal was reduced to 0.947 of the variance in the raw LFP (*P* = .003, jackknifed *t*-test), and the LFP cleaned by SUA3 was reduced to 0.928 of the raw variance (*P* = .002, jackknifed *t*-test).

The middle row of Figure 2(b) shows the effects of removing LFP signals that could be predicted by the MUA signal. As in the SUA examples, the LFP changed most dramatically during epochs of elevated spiking activity. The effect of removing the MUA-coupled signal was slightly, but not signficantly greater than SUA4 or SUA3 (jackknifed *t*-test), and the clean LFP was reduced to 0.899 of the power in the raw signal (*P* = .0008, jackknifed *t*-test).

Figure 3 illustrates the same procedure applied to a second recording site with weaker coupling between spikes and LFP. The impulse response for SUA and MUA spiking had smaller amplitude than for the previous example site (insets in Figures 3(a) and 3(b)). In this case removing components of the LFP that could be predicted by either the SUA or MUA had very little effect on the appearance of the LFP. Correspondingly, the power in the cleaned LFP signal was not significantly reduced from that of the raw LFP (jackknifed *t*-test, SUA4: 0.982, SUA3: 0.982, MUA: 0.981).

The examples in Figures 2 and 3 suggest that there was substantial variability in the portion of the LFP that could be explained by spiking activity, depending on the recording site. To study this effect across a larger set of recording sites, we measured the ratio of power (i.e., variance) in the clean and raw LFP signals for each recording site in the study (*n* = 127). Figure 4 plots histograms of the ratio for each of the three spike removal methods. For spike activity defined by SUA4, LFP power was reduced significantly for 58/127 sites (*P* < .05 jackknifed *t*-test), and the mean ratio of clean to raw LFP power was 0.956 (Figure 4(a)). The mean ratio for SUA3 (76/127 significantly reduced sites, *P* < .05) was 0.934, significantly lower than SUA4 (Figure 4(b), jackknifed *t*-test, *P* = .0008). The mean ratio for MUA (86/127 significantly reduced sites, *P* < .05) was 0.917, significantly lower than still than SUA3 (Figure 4(c), jackknifed *t*-test, *P* = .0007). On a site-by-site basis, the magnitude of SUA4-SUA3 decrease was correlated with the SUA3-MUA decrease (*r* = 0.66). The fact that SUA and MUA influences on the LFP covary suggests that the magnitude of spike-LFP correlation is a property of the site or the recording electrode.

**Figure 5:**
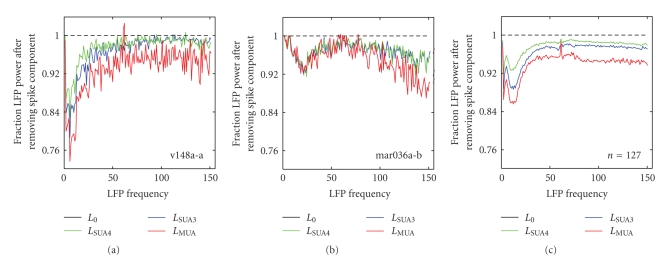
Frequency specificity of the spike-coupled LFP signal. (a) Relative power spectrum of the LFP signal from Figure 2 after removing SUA4, SUA3, and MUA components (colors as in previous figures). This site showed a large decrease at low frequencies (1–25 Hz). (b) Relative power spectrum of the LFP signal from the site in Figure 3 after the removal of spiking components (plotted as in *A*). This site showed a decrease near 25 Hz and at frequencies above 75 Hz. (c) Average relative power spectrum of LFP signal averaged across *n* = 127 recording sites. Removing coupled spike activity from the LFP signal reduced power at all frequencies. The effect was the strongest at low frequencies (1–10 Hz) but also showed a tendency to grow larger at high frequencies. Consistent with the overall changes in power reported in Figure 4, the LFP spectrum was reduced more for the more permissive definitions of spiking (MUA < SUA3 < SUA4). The small features around 60 Hz (most prominent for the signal with MUA components removed) reflect artifacts of line noise.

Because analysis of LFP often focuses on a specific spectral range of the LFP signal such as beta (20–30 Hz [7, 8]) or gamma (30–80 Hz, [10]), we wondered if removing spike information from the LFP signal affected some ranges of the spectrum more than others. To investigate this issue we compared the power spectrum of the LFP before and after removing spike-coupled components. Figure 5(a) shows the difference between the raw and clean LFP signals for the site shown in Figure 2. For this site, the reduction in LFP power was the greatest for frequencies below 20 Hz. The change in power also depended on the definition of spiking activity, with *L*_SUA4_ showing the smallest decrease and *L*_MUA_ showing the largest, following the pattern observed in the total power reduction (Figure 4).

Figure 5(b) shows the spectral analysis of changes in power for the site shown Figure 3. Here the change in power followed a different pattern. In this case, power was not reduced as much at lower frequencies. Instead, there was a reduction in a band around 25 Hz and also one that grew increasingly larger for frequencies above 75 Hz. As in the previous example, the reduction for MUA was larger than for the SUA signals.

The average change in LFP spectrum over the entire sample of recording sites is shown in Figure 5(c). Removing spike-coupled components reduced power at all frequencies. The reduction was the strongest at frequencies below 20 Hz, but it continued into the high gamma range, where most analyses of sensory tuning in the LFP have been performed [6, 10]. We were surprised to find large decreases power at low frequencies of the LFP. This may reflect large depolarization associated with periods of elevated spiking activity, but understanding this effect remains a direction for future investigation.

Of particular interest to studies that draw connections between single unit activity and large-scale measurements of neural activity is whether single units and LFP recorded at the same site are modulated in the same way by sensory stimuli. In A1, neurons are organized tonotopically [14, 18], meaning that nearby neurons tend to respond maximally to sounds (tones and narrowband noise) at the same best frequency (BF), and BF tends to change monotonically as the recording site is moved across A1. Because LFP is thought to represent the synaptic activity of neurons in the local anatomical area, the LFP is sometimes used to test if neighboring neurons do in fact encode the same sensory information. Previous reports have supported this idea, finding that the frequency tuning of single units and LFP is about the same [6, 11]. However, it is not clear how much LFP activity coupled with spiking by the small number of neurons closest to the recording site might bias LFP tuning measurements. 

**Figure 6:**
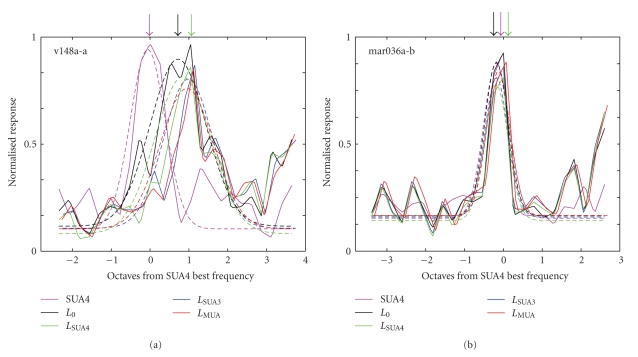
Effect of removing spike-correlated activity on the frequency tuning of LFP in A1. (a) Frequency tuning curves for site shown in Figure 2. Gaussian fits are plotted with dashed lines and best frequency (peak of the Gaussian fits) is indicated by arrows. The raw LFP tuning curve was centered at a higher best frequency than the SUA4 curve (0.92 octaves above SUA4, *P* = .007, jackknifed *t*-test). After the SUA4-coupled component was removed, the LFP tuning curve was shifted to even higher frequencies (1.63 octaves above SUA4, *P* = .01, jackknifed *t*-test). Similar curves to this last case are observed for the LFP signals with SUA3 and MUA components removed. (b) Tuning curves for site shown in Figure 3 (plotted as in *A*). For this site, there was no significant difference between the SUA and LFP tuning curves (<0.1 octave difference), even after the spike-coupled component was removed from the LFP.

We compared the auditory tuning of spiking activity, raw LFP and clean LFP in order to see if coupled spiking activity significantly influences tuning of the LFP signal. Responses were measured to band-pass noise stimuli centered at logarithmically spaced frequencies (see Methods for details). Figure 6(a) compares the tuning of the onset response of each of these signals for the site shown in Figure 2. The response of the SUA4 signal was measured from the average firing rate during 150 ms after stimulus onset, and the response of the LFP signals was measured by their standard deviation during the same 150 ms period. Baseline activity (absent any stimulus) was subtracted from each tuning curve. The SUA4 tuning curve was normalized to a maximum value of 1, and all the LFP signals were normalized by the same value so that the peak of the raw *L*_0_ signal also had a maximum of 1. Each tuning curve (solid line) was overlaid with a minimum mean-square error Gaussian fit (dashed line), whose parameters indicate basic tuning properties such as BF (mean of the Gaussian) and bandwidth (width of the Gaussian). Tuning curves were centered on the BF of the SUA4 signal. 

For this recording site, the SUA4 signal showed frequency tuning typical of a recording site in A1, responding only to stimuli within about half an octave of BF. The tuning of the raw LFP signal (black curve) was shifted toward higher frequencies, centered about one octave above the SUA4 tuning (*P* = .007, jackknifed *t*-test). When spiking activity was removed from the LFP, response amplitude decreased slightly, and the tuning curve shifted toward even higher frequencies. Tuning curves for LFP cleaned with all three methods were shifted about 0.5 octave above the SUA4 BF, significantly higher than the BF measured from the raw LFP (*P* = .01, jackknifed *t*-test). Because the tuning curve analysis is linear, the tuning of the removed LFP component is the difference between tuning of the raw and clean LFP signals.

Not all recording sites showed such dramatic differences in tuning. Figure 6(b) shows frequency tuning curves for the site shown in Figure 3. In this case, the tuning curves were similar for the spiking data and both the raw and cleaned LFP signals. All were centered at the BF of SUA4 activity and had a bandwidth of about one octave. Thus even when the components of the LFP directly coupled to spiking activity on a trial-by-trial basis were removed, the LFP at this site continued to have the same auditory tuning as the spiking activity.

## 4. Discussion

This study investigated the linear coupling of single unit spiking activity and local field potentials (LFPs) recorded from the same high-impedance electrodes in primary auditory cortex (A1). We observed that the spiking activity of just a few neurons near the recording site can sometimes explain a significant portion of variance in the LFP signal. We also demonstrated that our method of removing spike-correlated information can reveal different auditory tuning than tuning measured in the raw LFP signal. Analysis of larger neuronal populations using this method can explore the hypothesis that spiking activity sometimes biases measurements of the LFP recorded at the same location.

The linear filter used to remove spike activity was intended specifically to remove only the components of the low-pass LFP signal that were directly correlated with spike events recorded from the same electrode, independent of the concurrently presented auditory stimulus. These low frequency components were likely composed of slow currents associated with each action potential and the EPSPs that immediately preceded it. Thus what was effectively removed for each SUA/MUA event was a stereotyped, low-frequency modulation of the recorded electrical potential associated with each neuronal discharge. Synaptic potentials and spiking events of the larger neural population that were not tightly correlated with these nearby spikes were preserved in the LFP. In addition, synaptic potentials that arrived at nearby neurons but did not elicit spikes also remained in the LFP signal, and thus some bias may have persisted. Because of this partial removal, this filtering procedure would not be appropriate for any analysis that aims to entirely preserve or remove local EPSP activity in the LFP signal.

The SUA3/4 definition of spiking events was relatively permissive. While this threshold is used as a starting point for some spike sorting algorithms, a more conservative measure of single unit events (e.g., SUA5 or isolated spike events after sorting) may be substituted for the spike signal used in the analysis proposed here. The result will be to remove less power from the LFP, but it will remove only power that is correlated with the specified spiking events.

Previous studies have used similar spike versus LFP mapping procedures to characterize the functional relationship between these two signals [19], although they sought specifically to find common information in the two signals recorded from different electrodes and did not consider the possibility of spike bleed-through. A similar method has previously been used to remove spike-related activity from LFP signals [16], but the magnitude of the effect removing spike activity was not described. Additionally, this method does not account for autocorrelation in spike events in the filter estimate, which can lead to incomplete filtering (see Figure 1).

The method presented here assumed a stationary relationship between spiking activity and the LFP. Although substantial drift was not observed in the recordings analyzed for this study, this procedure could be adapted to use a dynamic, nonstationary filter, such as has been used in studies of stimulus encoding by sensory systems [20].

### 4.1. Implication for Neurophysiological Studies

Previous studies have suggested that the LFP is a valuable neurophysiological signal that can be used as a proxy measurement for large scale synchronous spiking activity [1, 5] and as a means for linking single neuron recordings to fMRI BOLD signals [2]. However, the biophysical processes that produce the LFP are not completely understood. To learn more about the LFP, it is useful to discriminate what components can be explained directly by other known signals such as single unit activity. If the activity of a single neuron or a small number of neurons contributes significantly to the LFP, then what appears to be synchronous activity may in fact be dominated by the activity of just those nearby neurons. The results of this study suggest that the LFP recorded from the same electrode as single-unit activity is sometimes strongly influenced by the activity of a small number of neurons. Possible bias from spiking activity should be considered in the analysis of LFP data in order to ascertain whether the properties of the LFP signal actually reflect the activity of a large group of anatomically neighboring neurons.

The spectral analysis of changes in LFP power (Figure 5) shows that removing coupled spike activity can affect the LFP across a wide range of frequencies. The total power in the LFP remains relatively high after removing the spike-coupled component (particularly at gamma frequencies, which have received the most attention in studies of sensory tuning [6, 10]). However, it is important to remember that the spiking activity often has robust sensory tuning, and removing that component can still have a significant effect on sensory tuning of the LFP signal. The potential for such an effect is illustrated in the tuning curves in Figure 6, which shows that removing LFP components correlated with spikes on a trial-by-trial basis can in fact change auditory tuning of the LFP. This result suggests that neurons in a region of A1 may not be as homogeneous in their tuning properties as would be concluded without accounting for the spike bias.

This study only investigated the tuning of transient, phase-locked responses to auditory stimuli. LFP signals also contain oscillations in the gamma range that are not phase-locked to stimulus onsets, but whose power may be modulated by the presence of a stimulus [10, 21]. It remains to be explored if the tuning of oscillatory components of the LFP can also be influenced by coupled spike activity. However, the broad-band decrease in LFP power suggests that tuning in the gamma frequency range can be influenced by coupled spike activity.

The same basic procedure can be used to remove other correlated activity from LFP signals, much in the same way that muscle artifacts are sometimes removed from EEG [22]. For example, if LFP is recorded during a behavior that introduces muscle artifacts to the LFP signal, a motor event signal can be substituted for the spike signal. The same procedure can then be used to measure a motor event-LFP filter and remove motor artifacts from the LFP before further analysis.
